# Does cycling to work benefit mental health? A natural experiment using linked administrative data.

**DOI:** 10.23889/ijpds.v7i3.1835

**Published:** 2022-08-25

**Authors:** Laurie Berrie, Zhiqiang Feng, Tom Clemens, Lee Williamson, Chris Dibben

**Affiliations:** 1 University of Edinburgh

## Objectives

We spend an increasing part of our daily lives commuting. Observational studies have shown commuting by bicycle improves subjective well-being. This study uses a quasi-experimental approach and an objective measure of mental health taken from linked administrative data to ask “does cycling to work benefit mental health?”

## Approach

Previous studies have tended to use cross-sectional survey data which have small sample sizes and self-reported health measures that are subject to reporting bias. The potential for unmeasured confounding and reverse causation also weakens any conclusions. Our study links the Scottish population census with NHS Prescribing Information System records for data on commuting in Edinburgh and Glasgow and mental health prescriptions, respectively. Although there are complexities in determining causal effects from observational data, we employ an instrumental variable approach to address this using road distance from home to nearest cycle path as an instrument.

## Results

Approximately 400,000 people aged 16-74 living and working in the City of Edinburgh and Glasgow City council areas as at the 2011 census were included in our study. Of those, 1.85% of commuters in Glasgow and 4.8% of commuters in Edinburgh cycled to work. Amongst cyclists 9% had a prescription for mental health compared to 14% amongst non-cyclists. More than 50% of commutes in both cities are less than 5km. Using the instrumental variable in a bivariate probit model we estimate an average treatment effect of -16.1% (95% confidence interval: -15.1% to -17.1%) in mental health prescriptions amongst those who cycle to work compared to those who use other forms of transport.

## Conclusion

This work confirms the benefit of cycling to work for mental health and provides further evidence in support of the promotion of active travel to encourage commuters travelling shorter distances to shift to cycling to work.

